# 1-[3-(Morpholin-4-yl)prop­yl]-3-[(naph­tha­len-2-yl)oxy]-4-(3-nitro­phen­yl)azeti­din-2-one

**DOI:** 10.1107/S1600536814014949

**Published:** 2014-07-02

**Authors:** Zeliha Atioğlu, Mehmet Akkurt, Aliasghar Jarrahpour, Roghayeh Heiran, Namık Özdemir

**Affiliations:** a Ilke Education and Health Foundation, Cappadocia Vocational College, The Medical Imaging Techniques Program, 50420 Mustafapaşa, Ürgüp, Nevşehir, Turkey; b Department of Physics, Faculty of Sciences, Erciyes University, 38039 Kayseri, Turkey; c Department of Chemistry, College of Sciences, Shiraz University, 71454 Shiraz, Iran; d Department of Physics, Faculty of Arts and Sciences, Ondokuz Mayıs University, 55139 Samsun, Turkey

**Keywords:** crystal structure

## Abstract

In the title compound, C_26_H_27_N_3_O_5_, the β-lactam (azetidin-2-one) ring is nearly planar [maximum deviation = 0.011 (3) Å]. The mean plane formed by the four C atoms of the morpholine ring, which adopts a chair conformation, the benzene ring and the naphthalene ring system form dihedral angles of 72.85 (17), 87.46 (15) and 65.96 (11)°, respectively, with the β-lactam ring. In the crystal, molecules are linked *via* C—H⋯O hydrogen bonds, forming inversion dimers with *R*_2_^2^(8).

## Related literature   

For general background to β-lactams, see: Mehta *et al.* (2010 ▶); Arumugam *et al.* (2011 ▶); Myangar & Raval (2012 ▶); Singh & Sudheesh (2014 ▶); Abdellaoui & Xu (2014 ▶); Cheng & Cheng (2007 ▶); Xiang (2013 ▶). For ring-puckering parameters, see: Cremer & Pople (1975 ▶). For hydrogen-bond motifs, see: Bernstein *et al.* (1995 ▶).
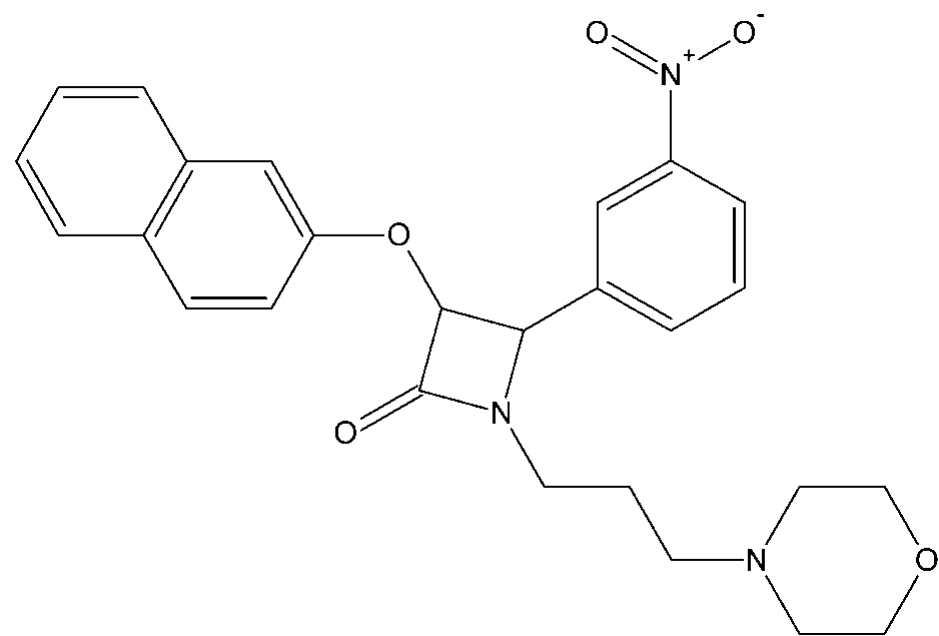


## Experimental   

### 

#### Crystal data  

C_26_H_27_N_3_O_5_
*M* *_r_* = 461.51
Triclinic, 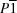

*a* = 9.7068 (8) Å
*b* = 10.3836 (9) Å
*c* = 14.2041 (11) Å
α = 73.739 (6)°
β = 75.922 (6)°
γ = 63.107 (6)°
*V* = 1214.33 (19) Å^3^
*Z* = 2
Mo *K*α radiation
μ = 0.09 mm^−1^
*T* = 296 K
0.51 × 0.39 × 0.25 mm

#### Data collection  

Stoe IPDS 2 diffractometer
Absorption correction: integration (*X-RED32*; Stoe & Cie, 2002 ▶) *T*_min_ = 0.967, *T*_max_ = 0.985
10059 measured reflections
4486 independent reflections
2123 reflections with *I* > 2σ(*I*)
*R*_int_ = 0.088

#### Refinement  

*R*[*F* ^2^ > 2σ(*F* ^2^)] = 0.066
*wR*(*F* ^2^) = 0.176
*S* = 0.95
4486 reflections
307 parameters
H-atom parameters constrained
Δρ_max_ = 0.28 e Å^−3^
Δρ_min_ = −0.17 e Å^−3^

### 

Data collection: *X-AREA* (Stoe & Cie, 2002 ▶); cell refinement: *X-AREA*; data reduction: *X-RED32* (Stoe & Cie, 2002 ▶); program(s) used to solve structure: *SHELXS2013* (Sheldrick, 2008 ▶); program(s) used to refine structure: *SHELXL2013* (Sheldrick, 2008 ▶); molecular graphics: *ORTEP-3 for Windows* (Farrugia, 2012 ▶); software used to prepare material for publication: *WinGX* (Farrugia, 2012 ▶).

## Supplementary Material

Crystal structure: contains datablock(s) global, I. DOI: 10.1107/S1600536814014949/hg5396sup1.cif

Structure factors: contains datablock(s) I. DOI: 10.1107/S1600536814014949/hg5396Isup2.hkl

Supporting information file. DOI: 10.1107/S1600536814014949/hg5396Isup3.cml

CCDC reference: 1010107

Additional supporting information:  crystallographic information; 3D view; checkCIF report

## Figures and Tables

**Table 1:** Hydrogen-bond geometry (Å, °)

*D*—H⋯*A*	*D*—H	H⋯*A*	*D*⋯*A*	*D*—H⋯*A*
C2—H2⋯O1^i^	0.98	2.46	3.229 (4)	135

Symmetry code: (i) 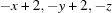.

**Fig. 1.**
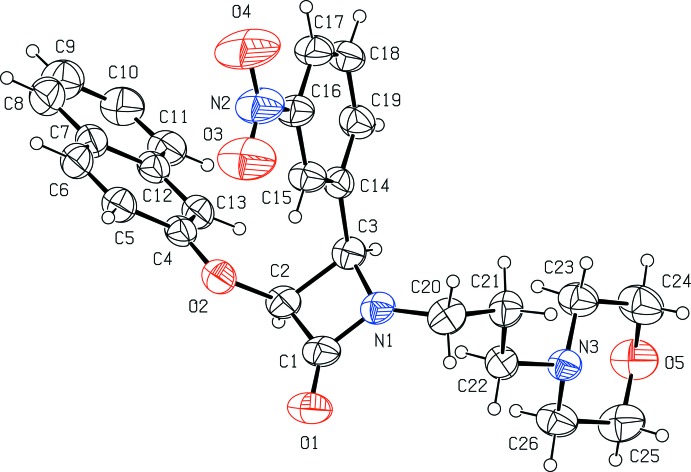
View of the molecular structure of (I) with the atom numbering scheme. Displacement ellipsoids for non-H atoms are drawn at the 30% probability level.

**Fig. 2.**
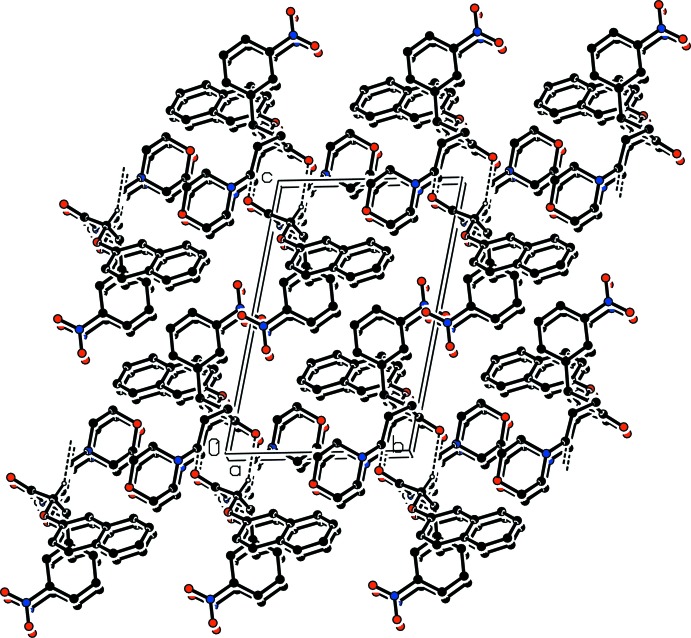
The packing and hydrogen bonding of (I) viewed along the a axis. Only H atoms involved in H bonding are shown.

## supplementary crystallographic information

### S1. Comment

The β-lactam ring is part of the core structure of most widely used antibiotics such as penicillins, cephalosporins, carbapenems, nocardicins and monobactam. Almost all of these antibiotics work by inhibiting bacterial cell wall biosynthesis (Mehta *et al.*, 2010; Arumugam *et al.*, 2011; Xiang, 2013; Myangar & Raval, 2012; Singh & Sudheesh, 2014). Functionalized β-lactams have attracted continued interests not only for their diverse and antibiotic activity, but also for their utility as versatile synthetic intermediates in organic synthesis as well as many other interesting biological properties (Cheng & Cheng, 2007; Abdellaoui & Xu, 2014). Therefore, there has been renewed interest in the synthesis of such interesting β-lactam based heterocycles with potential applications.

In the title compound (I, Fig. 1), the β-lactam ring (N1/C1–C3) is nearly planar, with the maximum deviations of -0.011 (2) Å for N1 and 0.011 (3) Å for C1 from the mean plane. The β-lactam ring makes dihedral angles of 72.85 (17), 87.46 (15) and 65.96 (11)°, respectively, with the least-squares plane formed by the four C atoms of the morpholine ring (N3/O5/C23–C26), the benzene ring (C14–C19), and the naphthalene ring system (C4–C13).

The morpholine ring adopts a chair conformation with puckering parameters: Q_T_ = 0.552 (4) Å, θ = 176.9 (4)° and φ = 44 (11)° (Cremer & Pople, 1975).

In the crystal structure, molecules are linked by pairs of weak C—H···O hydrogen bonds, forming inversion dimers, forming *R*_2_^2^(8) motifs (Bernstein *et al.*, 1995) along the [001] direction (Table 1, Fig. 2).

### S2. Experimental

A mixture of *N*-(3-nitrobenzylidene)-3-morpholinopropan-1-amine (1.38 g, 5.00 mmol) and triethylamine (2.53 g, 25.00 mmol), 2-naphthoxyacetic acid (1.54 g, 7.50 mmol) and tosyl chloride (1.43 g, 7.50 mmol) in CH_2_Cl_2_ (25 ml) was stirred at room temperature overnight. Then it was washed with HCl 1 *M* (20 ml), saturated NaHCO_3_ (20 ml) and brine (20 ml), dried over anhydrous Na_2_SO_4_ and the solvent was evaporated to give the crude product which was purified by column chromatography (eluent 10:1 EtOAc/EtOH) as off white crystals (yield 63%). mp: 399 - 401 K. IR (KBr, cm^-1^): 1759 (CO, β-lactam), 1350, 1527 (NO_2_). ^1^H-NMR (CDCl_3_) δ (p.p.m.): 1.72 (CH_2_—CH_2_—CH_2_–, m, 2H), 2.44 (CH_2_—CH_2_—CH_2_– and CH_2_—N morpholine ring, m, 6H), 3.02 (CH_2_—CH_2_—CH_2_–, m, 1H), 3.56 (CH_2_—CH_2_—CH_2_– and CH_2_—O morpholine ring, m, 5H), 5.18 (H-4, d, J = 4.4 Hz, 1H), 5.64 (H-3, d, J = 4.4 Hz, 1H), 8.84 (ArH, d, J = 8.9 Hz, 1H), 7.07 (ArH, s, 1H), 7.31–7.70 (ArH, m, 7H), 8.09 (ArH, d, J = 8.2 Hz, 1H), 8.24 (ArH, s, 1H). ^13^C-NMR (CDCl_3_) δ (p.p.m.): 24.4 (CH_2_—CH_2_—CH_2_–), 39.2 (CH_2_—CH_2_—CH_2_–), 53.5 (CH_2_—N morpholine ring), 56.0 (CH_2_—CH_2_—CH_2_–), 61.4 (C-4), 66.7 (CH_2_—O morpholine ring), 81.7 (C-3), 108.8, 117.9, 123.4, 123.8, 124.4, 126.6, 126.8, 127.6, 129.3, 129.5, 129.7, 133.8, 134.4, 135.8, 148.1, 154.2 (aromatic carbons), 165.5 (CO, β-lactam). MS m/*z* = 461 [*M*^+^]. Anal. Calcd. for C_26_H_27_N_3_O_5_: C 67.66, H 5.90, N 9.10%. Found: C 67.74, H 6.02, N 9.13%.

### S3. Refinement

H atoms were positioned geometrically and were refined using a riding model, with C—H = 0.93 - 0.98 Å, and *U*_iso_(H) = 1.2 *U*_eq_(C). Reflections (2 2 0), (2 0 2), (3 2 1) and (1 0 3) were omitted due to the large disagreement between F_obs_ and F_calc_. Due to weak diffracting ability of the crystal the ratio observed/unique reflections is low (47%). The unit cell contains a pair of voids of 44 Å^3^ about an inversion centre but the residual electron density (highest peak = 0.28 e Å^-3^ and deepest hole = -0.17 e Å^-3^) in the difference Fourier map suggests that no solvent molecule occupies this void.

### Crystal data

**Table:**

C_26_H_27_N_3_O_5_	*Z* = 2
*M**_r_* = 461.51	*F*(000) = 488
Triclinic, *P*1	*D*_x_ = 1.262 Mg m^−^^3^
Hall symbol: -P 1	Mo *K*α radiation, λ = 0.71073 Å
*a* = 9.7068 (8) Å	Cell parameters from 9755 reflections
*b* = 10.3836 (9) Å	θ = 1.5–28.8°
*c* = 14.2041 (11) Å	µ = 0.09 mm^−^^1^
α = 73.739 (6)°	*T* = 296 K
β = 75.922 (6)°	Block, light yellow
γ = 63.107 (6)°	0.51 × 0.39 × 0.25 mm
*V* = 1214.33 (19) Å^3^	

### Data collection

**Table:**

Stoe IPDS 2 diffractometer	4486 independent reflections
Radiation source: sealed X-ray tube, 12 x 0.4 mm long-fine focus	2123 reflections with *I* > 2σ(*I*)
Plane graphite monochromator	*R*_int_ = 0.088
Detector resolution: 6.67 pixels mm^-1^	θ_max_ = 25.5°, θ_min_ = 2.2°
ω scans	*h* = −11→11
Absorption correction: integration (*X-RED32*; Stoe & Cie, 2002)	*k* = −12→12
*T*_min_ = 0.967, *T*_max_ = 0.985	*l* = −17→16
10059 measured reflections	

### Refinement

**Table:**

Refinement on *F*^2^	0 restraints
Least-squares matrix: full	Hydrogen site location: inferred from neighbouring sites
*R*[*F*^2^ > 2σ(*F*^2^)] = 0.066	H-atom parameters constrained
*wR*(*F*^2^) = 0.176	*w* = 1/[σ^2^(*F*_o_^2^) + (0.0805*P*)^2^] where *P* = (*F*_o_^2^ + 2*F*_c_^2^)/3
*S* = 0.95	(Δ/σ)_max_ < 0.001
4486 reflections	Δρ_max_ = 0.28 e Å^−^^3^
307 parameters	Δρ_min_ = −0.17 e Å^−^^3^

### Special details

**Table:**

Geometry. Bond distances, angles *etc*. have been calculated using the rounded fractional coordinates. All su's are estimated from the variances of the (full) variance-covariance matrix. The cell e.s.d.'s are taken into account in the estimation of distances, angles and torsion angles
Refinement. Refinement on *F*^2^ for ALL reflections except those flagged by the user for potential systematic errors. Weighted *R*-factors *wR* and all goodnesses of fit *S* are based on *F*^2^, conventional *R*-factors *R* are based on *F*, with *F* set to zero for negative *F*^2^. The observed criterion of *F*^2^ > σ(*F*^2^) is used only for calculating -*R*-factor-obs *etc*. and is not relevant to the choice of reflections for refinement. *R*-factors based on *F*^2^ are statistically about twice as large as those based on *F*, and *R*-factors based on ALL data will be even larger.

### Fractional atomic coordinates and isotropic or equivalent isotropic displacement parameters (Å^2^)

**Table:**

	*x*	*y*	*z*	*U*_iso_*/*U*_eq_	
O1	0.9297 (3)	1.1424 (3)	0.07366 (17)	0.0961 (10)	
O2	0.7976 (2)	0.9168 (2)	0.21877 (14)	0.0753 (8)	
O3	0.8014 (5)	1.0509 (4)	0.4976 (2)	0.1465 (14)	
O4	0.8408 (5)	0.8838 (4)	0.6243 (2)	0.172 (2)	
O5	1.7676 (3)	0.5318 (3)	−0.1356 (2)	0.1070 (11)	
N1	1.1173 (3)	0.9410 (3)	0.16271 (17)	0.0677 (10)	
N2	0.8603 (5)	0.9237 (5)	0.5366 (2)	0.1089 (15)	
N3	1.5567 (3)	0.7649 (3)	−0.03474 (17)	0.0658 (9)	
C1	0.9845 (4)	1.0180 (4)	0.1211 (2)	0.0716 (11)	
C2	0.9402 (4)	0.8877 (4)	0.1546 (2)	0.0711 (11)	
C3	1.0932 (4)	0.8057 (3)	0.2041 (2)	0.0657 (11)	
C4	0.7483 (4)	0.8039 (4)	0.2538 (2)	0.0679 (11)	
C5	0.6102 (4)	0.8383 (4)	0.3220 (2)	0.0810 (11)	
C6	0.5486 (4)	0.7382 (5)	0.3597 (2)	0.0852 (14)	
C7	0.6183 (4)	0.5988 (4)	0.3316 (2)	0.0779 (13)	
C8	0.5543 (5)	0.4935 (6)	0.3687 (3)	0.0995 (18)	
C9	0.6232 (6)	0.3623 (6)	0.3379 (4)	0.114 (2)	
C10	0.7574 (6)	0.3308 (5)	0.2710 (3)	0.1057 (19)	
C11	0.8243 (5)	0.4271 (4)	0.2350 (3)	0.0871 (16)	
C12	0.7571 (4)	0.5656 (4)	0.2643 (2)	0.0723 (11)	
C13	0.8214 (4)	0.6705 (4)	0.2268 (2)	0.0715 (11)	
C14	1.0772 (3)	0.7610 (3)	0.3149 (2)	0.0618 (10)	
C15	0.9834 (4)	0.8625 (3)	0.3742 (2)	0.0703 (11)	
C16	0.9656 (4)	0.8152 (4)	0.4753 (2)	0.0739 (11)	
C17	1.0402 (5)	0.6725 (4)	0.5206 (3)	0.0847 (15)	
C18	1.1351 (4)	0.5718 (4)	0.4615 (3)	0.0848 (14)	
C19	1.1519 (4)	0.6162 (4)	0.3600 (2)	0.0763 (12)	
C20	1.2491 (4)	0.9733 (4)	0.1582 (2)	0.0791 (14)	
C21	1.4021 (4)	0.8613 (4)	0.1168 (2)	0.0746 (11)	
C22	1.4024 (4)	0.8513 (4)	0.0131 (2)	0.0718 (11)	
C23	1.6225 (4)	0.6141 (4)	0.0167 (3)	0.0847 (14)	
C24	1.7765 (5)	0.5274 (4)	−0.0364 (3)	0.1093 (17)	
C25	1.7052 (5)	0.6771 (4)	−0.1862 (3)	0.1020 (16)	
C26	1.5468 (4)	0.7661 (4)	−0.1358 (2)	0.0847 (13)	
H2	0.94510	0.84580	0.09940	0.0850*	
H3	1.17070	0.72420	0.17220	0.0790*	
H5	0.56200	0.92930	0.34080	0.0970*	
H6	0.45820	0.76100	0.40520	0.1030*	
H8	0.46420	0.51380	0.41450	0.1190*	
H9	0.57950	0.29410	0.36210	0.1370*	
H10	0.80300	0.24130	0.25030	0.1270*	
H11	0.91550	0.40250	0.19030	0.1040*	
H13	0.91410	0.64830	0.18340	0.0860*	
H15	0.93290	0.96150	0.34630	0.0850*	
H17	1.02720	0.64410	0.58910	0.1010*	
H18	1.18800	0.47360	0.49010	0.1020*	
H19	1.21530	0.54660	0.32100	0.0910*	
H20A	1.25690	0.97640	0.22430	0.0950*	
H20B	1.23130	1.07000	0.11720	0.0950*	
H21A	1.48590	0.88780	0.11710	0.0890*	
H21B	1.42240	0.76530	0.15940	0.0890*	
H22A	1.33220	0.80730	0.01490	0.0860*	
H22B	1.36270	0.95000	−0.02650	0.0860*	
H23A	1.55120	0.56930	0.02280	0.1020*	
H23B	1.63540	0.61160	0.08290	0.1020*	
H24A	1.85050	0.56620	−0.03630	0.1310*	
H24B	1.81480	0.42580	−0.00120	0.1310*	
H25A	1.69630	0.67850	−0.25300	0.1220*	
H25B	1.77550	0.72220	−0.19050	0.1220*	
H26A	1.50790	0.86680	−0.17250	0.1010*	
H26B	1.47430	0.72500	−0.13480	0.1010*	

### Atomic displacement parameters (Å^2^)

**Table:**

	*U*^11^	*U*^22^	*U*^33^	*U*^12^	*U*^13^	*U*^23^
O1	0.112 (2)	0.0782 (16)	0.0649 (14)	−0.0169 (14)	−0.0123 (13)	−0.0032 (12)
O2	0.0629 (14)	0.0846 (15)	0.0576 (12)	−0.0106 (12)	−0.0013 (10)	−0.0233 (11)
O3	0.178 (3)	0.114 (2)	0.082 (2)	−0.006 (2)	0.015 (2)	−0.0443 (18)
O4	0.246 (5)	0.162 (3)	0.0539 (17)	−0.057 (3)	0.026 (2)	−0.0303 (17)
O5	0.124 (2)	0.0752 (17)	0.0928 (19)	−0.0129 (15)	0.0003 (16)	−0.0356 (14)
N1	0.0710 (19)	0.0701 (16)	0.0528 (14)	−0.0232 (15)	0.0043 (13)	−0.0197 (12)
N2	0.136 (3)	0.110 (3)	0.0547 (19)	−0.033 (2)	0.0065 (18)	−0.0251 (17)
N3	0.0670 (17)	0.0656 (16)	0.0528 (13)	−0.0176 (12)	−0.0016 (12)	−0.0164 (11)
C1	0.084 (2)	0.074 (2)	0.0428 (15)	−0.0215 (19)	−0.0004 (16)	−0.0171 (15)
C2	0.071 (2)	0.086 (2)	0.0449 (15)	−0.0215 (17)	0.0014 (15)	−0.0230 (15)
C3	0.065 (2)	0.0658 (19)	0.0536 (16)	−0.0155 (15)	0.0038 (14)	−0.0231 (14)
C4	0.058 (2)	0.084 (2)	0.0472 (15)	−0.0167 (18)	−0.0084 (14)	−0.0122 (15)
C5	0.062 (2)	0.092 (2)	0.0609 (19)	−0.0081 (19)	0.0021 (16)	−0.0233 (17)
C6	0.059 (2)	0.115 (3)	0.0569 (19)	−0.020 (2)	0.0055 (16)	−0.0194 (19)
C7	0.060 (2)	0.105 (3)	0.0532 (18)	−0.022 (2)	−0.0069 (16)	−0.0142 (18)
C8	0.080 (3)	0.129 (4)	0.080 (2)	−0.040 (3)	−0.005 (2)	−0.017 (3)
C9	0.123 (4)	0.126 (4)	0.099 (3)	−0.059 (3)	−0.021 (3)	−0.012 (3)
C10	0.118 (4)	0.099 (3)	0.093 (3)	−0.039 (3)	−0.017 (3)	−0.016 (2)
C11	0.085 (3)	0.090 (3)	0.068 (2)	−0.019 (2)	−0.0080 (18)	−0.0204 (19)
C12	0.060 (2)	0.091 (2)	0.0507 (16)	−0.0171 (18)	−0.0105 (15)	−0.0134 (16)
C13	0.0550 (19)	0.087 (2)	0.0537 (17)	−0.0114 (18)	−0.0009 (14)	−0.0228 (16)
C14	0.0565 (18)	0.0656 (19)	0.0569 (16)	−0.0184 (15)	−0.0073 (14)	−0.0149 (14)
C15	0.082 (2)	0.0648 (19)	0.0496 (16)	−0.0171 (16)	−0.0044 (15)	−0.0167 (13)
C16	0.088 (2)	0.081 (2)	0.0501 (17)	−0.0328 (19)	−0.0065 (16)	−0.0150 (16)
C17	0.100 (3)	0.096 (3)	0.0556 (18)	−0.043 (2)	−0.0189 (19)	0.0003 (19)
C18	0.090 (3)	0.072 (2)	0.082 (2)	−0.029 (2)	−0.029 (2)	0.0073 (19)
C19	0.073 (2)	0.069 (2)	0.075 (2)	−0.0176 (17)	−0.0127 (17)	−0.0144 (16)
C20	0.092 (3)	0.086 (2)	0.0628 (19)	−0.036 (2)	0.0025 (17)	−0.0324 (16)
C21	0.074 (2)	0.097 (2)	0.0592 (18)	−0.0372 (19)	−0.0003 (16)	−0.0291 (16)
C22	0.065 (2)	0.086 (2)	0.0564 (17)	−0.0224 (17)	−0.0041 (15)	−0.0211 (15)
C23	0.094 (3)	0.076 (2)	0.070 (2)	−0.022 (2)	−0.0124 (19)	−0.0149 (17)
C24	0.108 (3)	0.080 (3)	0.103 (3)	−0.003 (2)	−0.017 (2)	−0.023 (2)
C25	0.123 (3)	0.085 (3)	0.071 (2)	−0.024 (2)	0.013 (2)	−0.0307 (19)
C26	0.095 (3)	0.082 (2)	0.0551 (18)	−0.0142 (19)	−0.0082 (17)	−0.0212 (15)

### Geometric parameters (Å, º)

**Table:**

O1—C1	1.213 (4)	C18—C19	1.378 (5)
O2—C2	1.414 (4)	C20—C21	1.516 (5)
O2—C4	1.384 (4)	C21—C22	1.505 (4)
O3—N2	1.200 (6)	C23—C24	1.493 (6)
O4—N2	1.194 (4)	C25—C26	1.510 (6)
O5—C24	1.419 (5)	C2—H2	0.9800
O5—C25	1.392 (5)	C3—H3	0.9800
N1—C1	1.351 (5)	C5—H5	0.9300
N1—C3	1.464 (4)	C6—H6	0.9300
N1—C20	1.446 (5)	C8—H8	0.9300
N2—C16	1.465 (5)	C9—H9	0.9300
N3—C22	1.463 (5)	C10—H10	0.9300
N3—C23	1.441 (5)	C11—H11	0.9300
N3—C26	1.458 (4)	C13—H13	0.9300
C1—C2	1.519 (6)	C15—H15	0.9300
C2—C3	1.566 (6)	C17—H17	0.9300
C3—C14	1.501 (4)	C18—H18	0.9300
C4—C5	1.411 (5)	C19—H19	0.9300
C4—C13	1.358 (5)	C20—H20A	0.9700
C5—C6	1.349 (6)	C20—H20B	0.9700
C6—C7	1.421 (6)	C21—H21A	0.9700
C7—C8	1.411 (7)	C21—H21B	0.9700
C7—C12	1.409 (5)	C22—H22A	0.9700
C8—C9	1.365 (8)	C22—H22B	0.9700
C9—C10	1.376 (8)	C23—H23A	0.9700
C10—C11	1.350 (7)	C23—H23B	0.9700
C11—C12	1.422 (5)	C24—H24A	0.9700
C12—C13	1.410 (6)	C24—H24B	0.9700
C14—C15	1.380 (4)	C25—H25A	0.9700
C14—C19	1.377 (5)	C25—H25B	0.9700
C15—C16	1.376 (4)	C26—H26A	0.9700
C16—C17	1.362 (5)	C26—H26B	0.9700
C17—C18	1.375 (6)		
			
C2—O2—C4	117.4 (3)	C14—C3—H3	112.00
C24—O5—C25	110.2 (3)	C4—C5—H5	120.00
C1—N1—C3	96.4 (3)	C6—C5—H5	120.00
C1—N1—C20	132.1 (3)	C5—C6—H6	119.00
C3—N1—C20	131.1 (3)	C7—C6—H6	119.00
O3—N2—O4	121.9 (4)	C7—C8—H8	120.00
O3—N2—C16	119.0 (3)	C9—C8—H8	120.00
O4—N2—C16	119.0 (4)	C8—C9—H9	120.00
C22—N3—C23	112.9 (3)	C10—C9—H9	120.00
C22—N3—C26	109.7 (3)	C9—C10—H10	119.00
C23—N3—C26	108.5 (3)	C11—C10—H10	119.00
O1—C1—N1	131.9 (4)	C10—C11—H11	119.00
O1—C1—C2	136.2 (4)	C12—C11—H11	120.00
N1—C1—C2	91.9 (3)	C4—C13—H13	120.00
O2—C2—C1	113.2 (3)	C12—C13—H13	120.00
O2—C2—C3	116.9 (2)	C14—C15—H15	121.00
C1—C2—C3	85.7 (3)	C16—C15—H15	120.00
N1—C3—C2	85.9 (2)	C16—C17—H17	121.00
N1—C3—C14	115.7 (3)	C18—C17—H17	121.00
C2—C3—C14	117.7 (3)	C17—C18—H18	120.00
O2—C4—C5	114.0 (3)	C19—C18—H18	120.00
O2—C4—C13	125.1 (3)	C14—C19—H19	119.00
C5—C4—C13	120.9 (4)	C18—C19—H19	119.00
C4—C5—C6	119.6 (4)	N1—C20—H20A	109.00
C5—C6—C7	121.6 (4)	N1—C20—H20B	109.00
C6—C7—C8	122.6 (4)	C21—C20—H20A	109.00
C6—C7—C12	118.1 (4)	C21—C20—H20B	109.00
C8—C7—C12	119.3 (4)	H20A—C20—H20B	108.00
C7—C8—C9	120.6 (5)	C20—C21—H21A	109.00
C8—C9—C10	120.1 (5)	C20—C21—H21B	109.00
C9—C10—C11	121.4 (5)	C22—C21—H21A	109.00
C10—C11—C12	120.9 (4)	C22—C21—H21B	109.00
C7—C12—C11	117.7 (4)	H21A—C21—H21B	108.00
C7—C12—C13	119.6 (3)	N3—C22—H22A	109.00
C11—C12—C13	122.7 (4)	N3—C22—H22B	109.00
C4—C13—C12	120.2 (3)	C21—C22—H22A	109.00
C3—C14—C15	121.1 (3)	C21—C22—H22B	109.00
C3—C14—C19	120.7 (3)	H22A—C22—H22B	108.00
C15—C14—C19	118.2 (3)	N3—C23—H23A	109.00
C14—C15—C16	119.1 (3)	N3—C23—H23B	109.00
N2—C16—C15	118.2 (3)	C24—C23—H23A	109.00
N2—C16—C17	118.8 (3)	C24—C23—H23B	109.00
C15—C16—C17	123.1 (3)	H23A—C23—H23B	108.00
C16—C17—C18	117.8 (4)	O5—C24—H24A	109.00
C17—C18—C19	120.1 (4)	O5—C24—H24B	109.00
C14—C19—C18	121.7 (3)	C23—C24—H24A	109.00
N1—C20—C21	113.1 (3)	C23—C24—H24B	109.00
C20—C21—C22	112.8 (3)	H24A—C24—H24B	108.00
N3—C22—C21	113.6 (3)	O5—C25—H25A	109.00
N3—C23—C24	111.5 (3)	O5—C25—H25B	109.00
O5—C24—C23	112.4 (4)	C26—C25—H25A	109.00
O5—C25—C26	112.0 (3)	C26—C25—H25B	109.00
N3—C26—C25	110.4 (3)	H25A—C25—H25B	108.00
O2—C2—H2	113.00	N3—C26—H26A	109.00
C1—C2—H2	113.00	N3—C26—H26B	110.00
C3—C2—H2	113.00	C25—C26—H26A	110.00
N1—C3—H3	112.00	C25—C26—H26B	110.00
C2—C3—H3	112.00	H26A—C26—H26B	108.00
			
C2—O2—C4—C5	176.4 (3)	C2—C3—C14—C15	−54.0 (5)
C4—O2—C2—C1	−179.5 (3)	N1—C3—C14—C19	−136.8 (4)
C4—O2—C2—C3	−82.2 (4)	O2—C4—C13—C12	−177.4 (3)
C2—O2—C4—C13	−4.1 (5)	C5—C4—C13—C12	2.1 (5)
C25—O5—C24—C23	55.7 (5)	C13—C4—C5—C6	−1.1 (5)
C24—O5—C25—C26	−56.8 (5)	O2—C4—C5—C6	178.5 (3)
C20—N1—C1—C2	171.6 (3)	C4—C5—C6—C7	−0.8 (5)
C3—N1—C1—O1	179.9 (4)	C5—C6—C7—C12	1.5 (5)
C20—N1—C1—O1	−6.8 (6)	C5—C6—C7—C8	−178.8 (4)
C3—N1—C20—C21	50.6 (4)	C12—C7—C8—C9	−1.8 (7)
C20—N1—C3—C14	69.3 (4)	C8—C7—C12—C13	179.9 (4)
C1—N1—C3—C2	1.7 (2)	C6—C7—C8—C9	178.5 (4)
C1—N1—C20—C21	−120.6 (4)	C6—C7—C12—C11	−178.7 (3)
C3—N1—C1—C2	−1.7 (2)	C6—C7—C12—C13	−0.4 (5)
C1—N1—C3—C14	−117.2 (3)	C8—C7—C12—C11	1.5 (5)
C20—N1—C3—C2	−171.8 (3)	C7—C8—C9—C10	0.8 (8)
O3—N2—C16—C17	175.7 (5)	C8—C9—C10—C11	0.5 (8)
O4—N2—C16—C17	−1.2 (8)	C9—C10—C11—C12	−0.7 (7)
O3—N2—C16—C15	−4.8 (7)	C10—C11—C12—C7	−0.3 (6)
O4—N2—C16—C15	178.3 (5)	C10—C11—C12—C13	−178.6 (4)
C26—N3—C22—C21	−177.8 (3)	C7—C12—C13—C4	−1.4 (5)
C23—N3—C26—C25	−56.2 (4)	C11—C12—C13—C4	176.9 (4)
C22—N3—C26—C25	−179.9 (3)	C3—C14—C19—C18	−178.0 (4)
C22—N3—C23—C24	177.4 (3)	C19—C14—C15—C16	−1.3 (6)
C26—N3—C23—C24	55.6 (4)	C3—C14—C15—C16	176.5 (4)
C23—N3—C22—C21	61.1 (4)	C15—C14—C19—C18	−0.1 (6)
O1—C1—C2—O2	−62.7 (5)	C14—C15—C16—C17	2.0 (7)
N1—C1—C2—O2	119.1 (3)	C14—C15—C16—N2	−177.4 (4)
N1—C1—C2—C3	1.6 (2)	N2—C16—C17—C18	178.2 (4)
O1—C1—C2—C3	179.8 (4)	C15—C16—C17—C18	−1.2 (7)
O2—C2—C3—C14	1.6 (4)	C16—C17—C18—C19	−0.3 (7)
C1—C2—C3—C14	115.5 (3)	C17—C18—C19—C14	0.9 (7)
O2—C2—C3—N1	−115.4 (3)	N1—C20—C21—C22	60.0 (4)
C1—C2—C3—N1	−1.5 (2)	C20—C21—C22—N3	169.3 (3)
C2—C3—C14—C19	123.8 (4)	N3—C23—C24—O5	−56.3 (5)
N1—C3—C14—C15	45.4 (5)	O5—C25—C26—N3	58.4 (5)

### Hydrogen-bond geometry (Å, º)

**Table:**

*D*—H···*A*	*D*—H	H···*A*	*D*···*A*	*D*—H···*A*
C2—H2···O1^i^	0.98	2.46	3.229 (4)	135

Symmetry code: (i) −*x*+2, −*y*+2, −*z*.
